# Protein Farnesylation Takes Part in Arabidopsis Seed Development

**DOI:** 10.3389/fpls.2021.620325

**Published:** 2021-01-28

**Authors:** Valentin Vergès, Christelle Dutilleul, Béatrice Godin, Boris Collet, Alain Lecureuil, Loïc Rajjou, Cyrille Guimaraes, Michelle Pinault, Stéphane Chevalier, Nathalie Giglioli-Guivarc’h, Eric Ducos

**Affiliations:** ^1^Biomolécules et Biotechnologies Végétales, Faculté de Pharmacie, Université de Tours, Tours, France; ^2^Institut Jean-Pierre Bourgin, INRA, AgroParisTech, Université Paris-Saclay, Versailles, France; ^3^Nutrition, Croissance et Cancer, INSERM UMR 1069, Université de Tours, Tours, France

**Keywords:** ERA1, protein farnesylation, plant reproduction, fatty acid, gynoecium, pollination

## Abstract

Protein farnesylation is a post-translational modification regulated by the *ERA1* (Enhanced Response to ABA 1) gene encoding the β-subunit of the protein farnesyltransferase in Arabidopsis. The *era1* mutants have been described for over two decades and exhibit severe pleiotropic phenotypes, affecting vegetative and flower development. We further investigated the development and quality of *era1* seeds. While the *era1* ovary contains numerous ovules, the plant produces fewer seeds but larger and heavier, with higher protein contents and a modified fatty acid distribution. Furthermore, *era1* pollen grains show lower germination rates and, at flower opening, the pistils are immature and the ovules require one additional day to complete the embryo sac. Hand pollinated flowers confirmed that pollination is a major obstacle to *era1* seed phenotypes, and a near wild-type seed morphology was thus restored. Still, *era1* seeds conserved peculiar storage protein contents and altered fatty acid distributions. The multiplicity of *era1* phenotypes reflects the diversity of proteins targeted by the farnesyltransferase. Our work highlights the involvement of protein farnesylation in seed development and in the control of traits of agronomic interest.

## Introduction

Post-translational processes are commonly used to control activity, stability, sub-cellular localization and/or protein interaction through modifications of biochemical properties of proteins. Among them protein isoprenylation is widely distributed in Eukaryotes and relies on the covalent attachment of a C15 farnesyl or a C20 geranylgeranyl isoprenoid onto a specific CaaX-box located at the C-terminal end of polypeptide chains (Zhang and Casey, 1996; Galichet and Gruissem, 2003; Hemsley, 2015). This is mediated by the CaaX-protein prenyltransferases (CaaX-PTases) (i.e., protein farnesyltransferase (PFT) and protein geranylgeranyltransferase type I (PGGT-I)). Both enzymes are heterodimers composed of a common α-subunit and a specific β-subunit (Lane and Beese, 2006). The identification of targets of CaaX-PTases is still poorly documented in plants. Among the 120 putative farnesylatable proteins predicted *in silico* in the *Arabidopsis thaliana* genome (Galichet and Gruissem, 2003), less than 15 have been functionally characterized. These proteins have wide-ranging functions, being involved in chaperone pathways, in phytohormone biosynthesis, in ubiquitin-mediated protein degradation or in gene expression regulation (Barth et al., 2009; Crowell and Huizinga, 2009; Dutilleul et al., 2016; Northey et al., 2016; Barghetti et al., 2017; Sjögren et al., 2018). For most of them, isoprenylation results in membrane anchoring or in modified subcellular location, promoting or not interactions with other proteins (Running, 2014; Hemsley, 2015).

The biological relevance of CaaX-protein isoprenylation in plant has been uncovered 20 years ago through the study of mutants compromised in one or both CaaX-PTases activities. Studies dedicated to Arabidopsis *ggb* (*geranylgeranyltransferase β-subunit*) mutant characterization highlighted few developmental phenotypes but suggested PGGT-I involvement in phytohormone signaling and in some aspects of plant defense (Johnson et al., 2005; Goritschnig et al., 2008). The Arabidopsis *era1* (enhanced response to abscisic acid1) mutant, impaired in the β-subunit of PFT, has been extensively studied due to its multifaceted phenotypes. *era1* has been discovered in a genetic screen designed to isolate mutants with altered ABA sensitivity at germination (Cutler et al., 1996). *era1*-mediated ABA sensitivity have been also associated to stomatal movements and to the resistance to hydric stress (Pei et al., 1998). Since this phenotype relies on traits of agronomic interests, numerous works have been undertaken to better understand the link between ABA and protein farnesylation. Hence, *era1*-mediated drought tolerance and increased seed dormancy are observed in crops such as wheat and rapeseed, as well (Wang et al., 2009; Manmathan et al., 2013). Several developmental defects characterize *era1* plants that display increased seed dormancy, delayed growth, longer lifespan, enlarged meristems, altered phyllotaxis and root architecture, as well as supernumerary floral organs (Cutler et al., 1996; Running et al., 1998, 2004; Yalovsky et al., 2000b; Ziegelhoffer et al., 2000; Brady et al., 2003). In addition, *era1* displays an enhanced susceptibility to pathogens and an increased tolerance to heat, indicating that farnesylation also participates to plant responses to both biotic and abiotic stress (Goritschnig et al., 2008; Wu et al., 2017).

The *era1* mutant has been extensively described through ABA signaling and for its developmental phenotypes, ranging from seed germination to flower morphology. According to the Arabidopsis eFP Browser atlas^1^ *ERA1* (At5g40280) is widely expressed in plant (data not shown). With *in situ* hybridization studies, Ziegelhoffer et al. (2000) revealed that *ERA1* is expressed during flower and embryo developments. Nevertheless, *era1* seed features (i.e., morphology and content) have not been documented so far, while ABA signaling largely contributes to seed development (Baud et al., 2008). Seed development is a complex process that starts by embryo morphogenesis and then proceeds by seed maturation during which accumulation of storage compounds occurs in the embryo. Many seed traits are also heavily dependent on ovule development maternal traits, especially those that have maternal sporophytic origin (Baud and Lepiniec, 2010; Nakashima and Yamaguchi-Shinozaki, 2013; Shu et al., 2016). In this complex process, ABA acts upstream of key transcriptional factors that control a wide range of seed-specific traits and play a critical role in seed quality, including lipid and protein contents (Baud et al., 2008). Among these, ABI3 (Abscisic acid Insensitive 3) is a major actor of the ABA-dependent seed maturation process and it participates in the control of storage compound biosynthesis (Giraudat et al., 1992; Roscoe et al., 2015). ABI3 acts downstream of ERA1 in the seed ABA responsiveness (Brady et al., 2003). Thus, one would expect *era1* plants to be affected in the seed maturation process.

In the present work, we characterized the seeds produced by Arabidopsis *era1-8* and *ggb-2* mutants. Our data show that protein farnesylation, but not geranylgeranylation, is engaged in seed size determination and in the production of seed storage compounds (i.e., protein and lipid contents). Moreover, ω3 and ω6 fatty acid (FA) repartition is altered in *era1* seeds. Further investigation linked the *era1* low seed production to an inefficient self-pollination and explained some *era1* seed peculiarities. Hence, our results reveal the functional plurality of protein farnesylation in the control of flower and seed development.

## Results

### *era1-8* Mutant, But Not *ggb-2* Mutant, Produces Larger and Heavier Seeds

**FIGURE 1:**
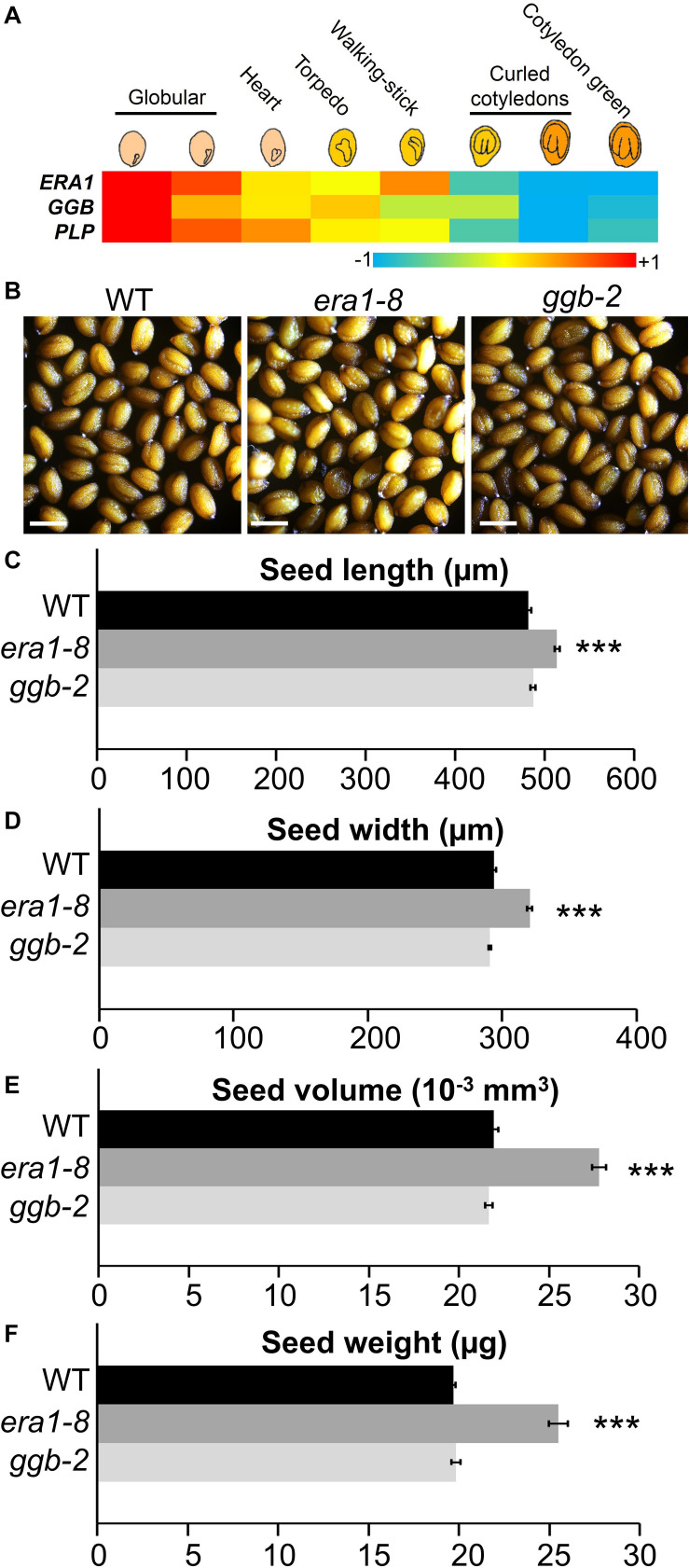
Seed morphological features of Arabidopsis prenylation mutants. **(A)** Heatmap of *ERA1*, *GGB* and *PLP* gene expression during seed development (data collected from Winter et al., 2007). **(B)** Representative mature seeds from WT, *era1-8 and ggb-2 mutants.* Scale bar, 500 μm. **(C)** and **(D)** Length and width correspond to an average of >250 measurements (i.e., >50 seeds from 5 independent biological replicates for each genotype) using ImageJ software and microscopy pictures. **(E)** The volume was calculated according to Riefler et al. (2006) using **(C)** and **(D)** data. **(F)** Seed mass was estimated by weighting 500 seeds of five different plants for each genotype with three technical replicates. Data represent mean ± SE. ***p-value < 0.001 (Student’s *t*-test).

**FIGURE 2:**
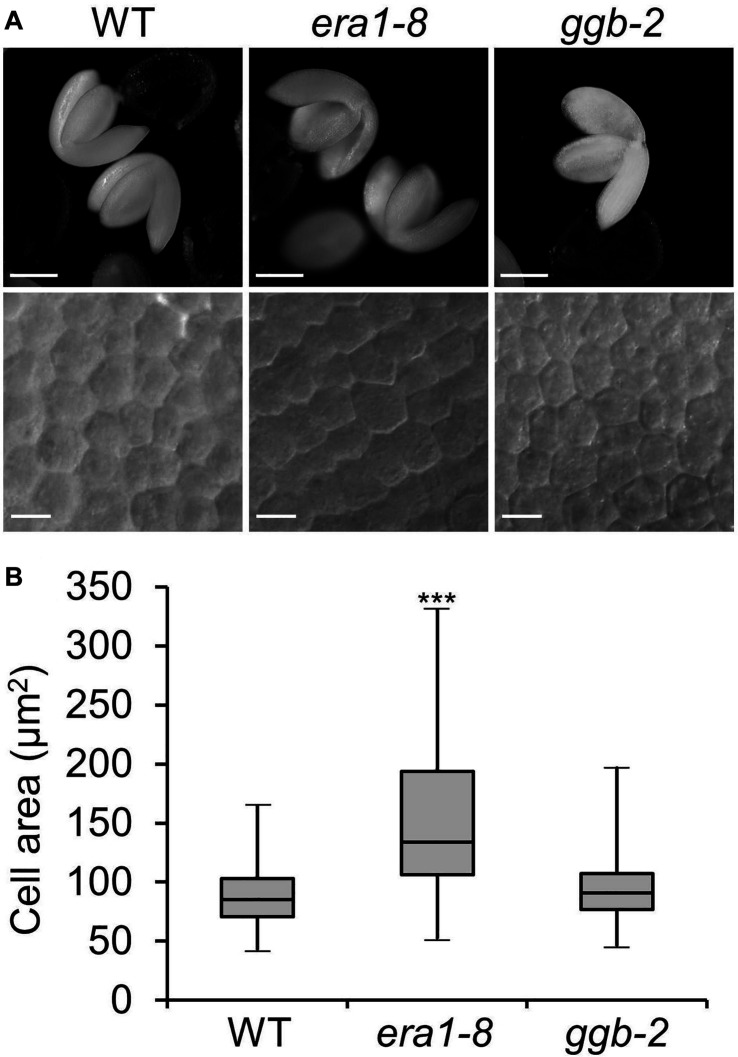
Embryo cell size. **(A)** Representative pictures of embryo (up) and embryo cells (down) from WT, *era1-8* and *ggb-2.* **(B)** Box-plot of embryo cell areas from WT, *era1-8* and *ggb-2.* Cell surface embryo were measured using differential interference contrast microscopy pictures and ImageJ software. Gray boxes represent 50% of measured cell areas, horizontal lines are the medians, the top and the bottom whiskers represent 25% of the measurements (*n* > 200). Scale bar, 250 μm for embryo pictures and 10 μm for embryo cells. ^∗∗∗^*P* value < 0.001 (Student’s *t*-test).

An heapmap generated by recovering the expression pattern of *ERA1*, *GGB* and *PLP* genes during eight stages of seed maturation (Winter et al., 2007) reveals that the genes encoding the subunits of protein isoprenylation enzymes are expressed during the early stages of seed development, then decrease until the green cotyledon stage (Figure 1A). In order to assess the impact of protein farnesylation or geranylgeranylation on seed production, we first investigated the seed production of the protein isoprenylation mutants *era1-8* (Goritschnig et al., 2008) and *ggb-2* (Running et al., 2004). Plants were grown under short day conditions and, as previously described (Bonetta et al., 2000), under these growth conditions, *era1* plants showed severe developmental phenotypes, whereas *ggb* plants developed similarly to WTs, without any obvious developmental phenotype. At first sight, isoprenylation mutants do not produce altered seeds in morphology nor color (Figure 1B). Nevertheless, while *era1-8* plants appear to be in poor shape, seed size measurements revealed that *era1-8* seeds are significantly longer (Figure 1C) and wider (Figure 1D) than WT. The inferred volume confirms that *era1-8* seeds are larger than those of the WT by 25% (Figure 1E). The size phenotype is supported by an increased seed weight in *era1-8*, by 25% too (Figure 1F). No difference could be detected for *ggb-2* seeds based on these criteria. A close-up view to embryo cells highlights that the surface of embryo cells increases in *era1-8* (Figure 2). Although seed size also relies on the size of the tegument cells, as well as endosperm development, the increased seed size of *era1-8* may be associated to bigger embryo cells in this mutant. Enlarged meristems have been already reported in *era1* and it is related to increased cell size too (Running et al., 1998; Yalovsky et al., 2000b). Hence, the farnesylation-dependent mechanisms that control meristem cell size might be extended to embryo cells.

### Near-Infrared Spectroscopy to Assess Isoprenylation Mutant Seed Content

**FIGURE 3:**
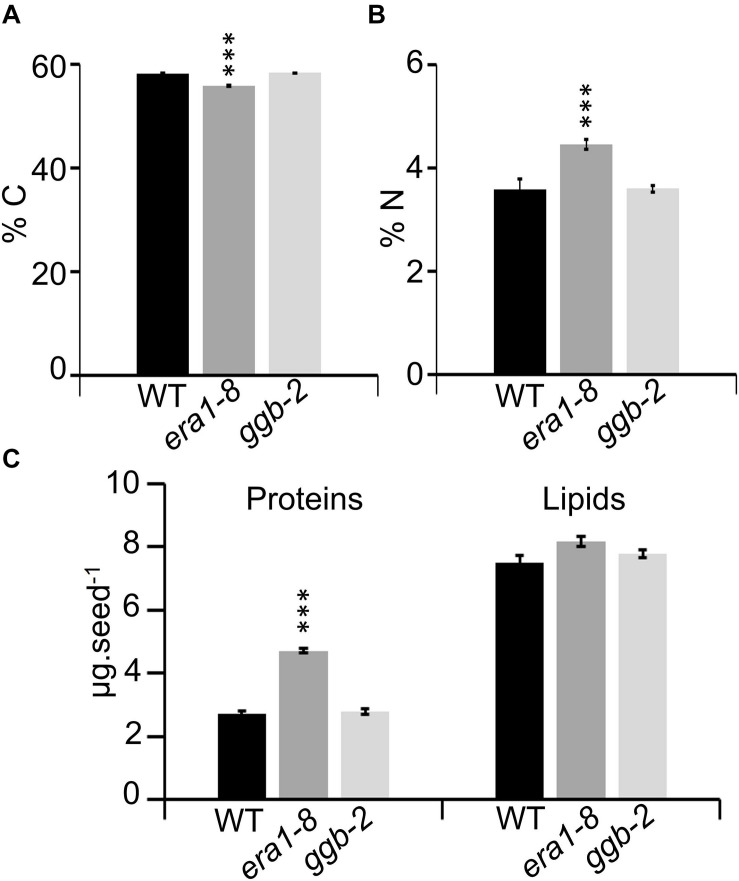
Near-infrared spectroscopy to assess seed carbon, nitrogen, protein and lipid contents. Graphs showing carbon **(A)** and nitrogen **(B)** contents of WT, *era1-8*, *ggb-2* seeds. **(C)** Graphs showing predicted seed protein and lipid contents (μg) expressed per seed. Data represent mean ± SE. ***p-value < 0.001 (Student’s *t*-test).

Near-infrared spectroscopy (NIRS) is a nondestructive and accurate method developed to assess carbon, nitrogen, lipid and protein in Arabidopsis seeds and allows the detection of seed filling modifications (Jasinski et al., 2016). NIRS quantification reveals that *era1-8* seeds contain a lower percentage of carbon and a higher percentage of nitrogen compared to WT and *ggb-2* (Figures 3A,B). According to NIRS predictive equations (Jasinski et al., 2016), *era1-8* seeds look enriched in proteins and depleted in lipids (Supplementary Figure 2A), nevertheless, when considering a single seed (heavier in *era1-8*, Figure 1F), lipid content is comparable in the three genotypes whereas protein content is higher in *era1-8* than in WT and *ggb-2* (Figure 3C). This suggests that *era1-8* seed weight is increased by an extra protein filling while the overall lipid quantity is not altered in this mutant.

### Protein Farnesylation Defect Alters Seed Protein Content

**FIGURE 4:**
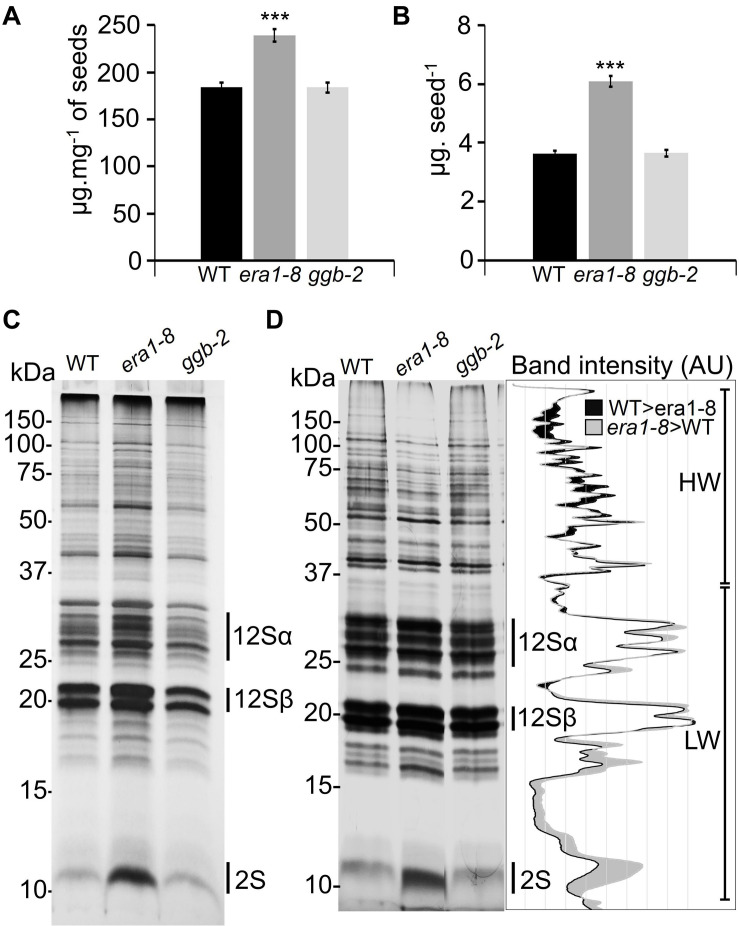
Qualitative analysis of protein contents in Arabidopsis prenylation mutant seeds. Quantification of total protein extracts from mature seeds expressed **(A)** as μg mg^– 1^ of seeds or **(B)** as μg seed^– 1^ using Bradford’s method (1976). Data present mean ± SE of 5 replicates. *** indicates a *p*-value < 0.001 (Student’s *t*-test). **(C)** SDS-PAGE loaded with the amount of proteins equivalent to one seed (silver nitrate staining), 12Sα and 12Sβ correspond to globulins, 2S corresponds to albumins. **(D)** SDS-PAGE loaded with 5 μg of total seed protein in each lane (silver nitrate staining). The graph on the right corresponds to WT and *era1-8* ImageJ plot profiles. HW and LW correspond to high-weight (>37 kDa) and low-weight (<37 kDa) proteins, respectively [according to Di Berardino et al. (2018)].

NIRS protein quantification was further strengthened by Bradford protein assays (Bradford, 1976) and confirmed that the *era1-8* seeds accumulate more proteins than WT and *ggb-2* (Figures 4A,B). Arabidopsis seeds contain two predominant types of storage proteins, 12S globulins and 2S albumins (Heath et al., 1986). They represent more than 80% of total seed proteins (Higashi et al., 2006) and constitute the main source of nitrogen and sulfur during the seed germination (Tabe et al., 2002). When a quantity of protein equivalent to one seed is separated on SDS-PAGE, *era1-8* displays a global pattern with more intense bands than WT and *ggb-2* (Figure 4C). When the same amount of protein (i.e., 5 μg) is loaded in each lane, all three patterns appear more balanced (Figure 3D). Because *era1-8* produces larger and heavier seeds, we could expect higher protein content in these seeds, but 1 mg of *era1-8* seeds contains more protein than 1 mg of WT (and *ggb-2*) seeds (Figures 3C, 4A), which would mean that *era1-8* seeds have somehow enriched protein content. Quantification of the band intensities performed after gel scanning (Di Berardino et al., 2018) shows that high molecular weight proteins (HW, above 37 kDa) are more abundant in WT than *era1-8* seeds, while low molecular-weight proteins (LW, below 37 kDa, mainly 12Sα and 12Sβ globulins and 2S albumins) are more abundant in *era1-8* than in WT seeds (Figure 4D, graph), especially the lowest 2S albumin band. Beside an increased seed size that accumulates more protein in seed, those results indicate that storage protein profiles is altered in *era1-8* and it affects 2S albumins rather than 12S globulins.

### Quantitative and Qualitative Analyses of Seed Lipids in the Protein Isoprenylation Mutants

**FIGURE 5:**
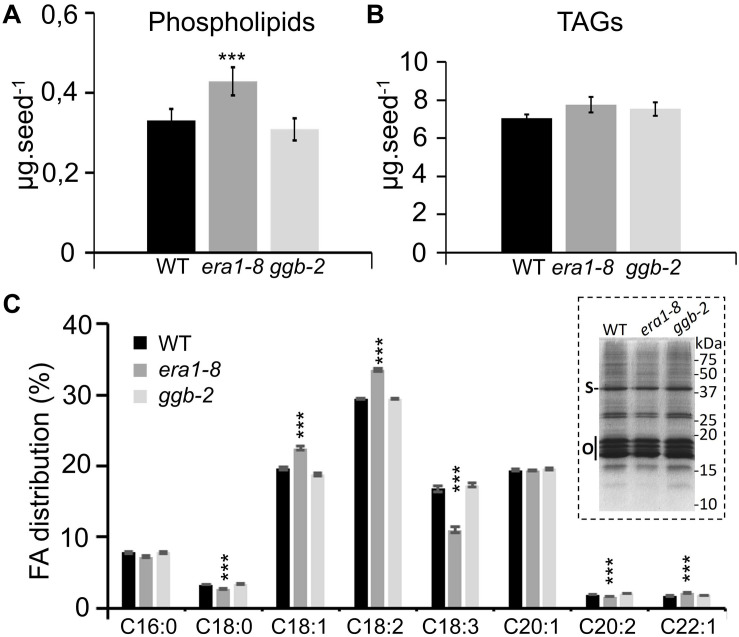
Comparison of lipid contents in WT, *era1-8* and *ggb-2* seeds. **(A)** Total phospholipids contents per seed. **(B)** TAG contents per seed. **(C)** FAs distribution in seeds (%). Inset shows lipid body protein patterns prepared from 25 mg of dry seeds (see section “Materials and Methods”); S, steroleosin and O, oleosins [according to Jolivet et al. (2004)]. Values are the mean ± SE of five independent replicates each composed of 10 mg of seeds. *** indicates a *p*-value < 0,001 (Student’s *t*-test).

Near-infrared spectroscopy experiments revealed comparable lipid contents in the seeds of the three genotypes (Figure 3C) but this approach cannot distinguish the different forms of lipids. So, to complement the NIRS data, seed lipid compositions were investigated through HPTLC analyses. WT, *era1-8* and *ggb-2* contain comparable quantities of phospholipids per mg of seeds (Supplementary Figure 2B), but individual seeds of *era1-8* display 30% more phospholipids (Figure 5A). This is consistent with the larger size of seeds and embryo cells observed in *era1-8.* Since Arabidopsis is an oleaginous plant, carbon reserves are mainly stored as triacylglycerols (TAGs) in specific lipid bodies of embryo cells (Murphy, 1993). TAGs consist of a glycerol bound to three fatty acids (FAs) and represent more than 90% of seed total lipids in Arabidopsis (Baud et al., 2008). So, we assume that NIRS analyses reflect TAG contents (7–7.9 μg per seed, Figure 5B) rather than minor seed lipids such as phospholipids (3.7–4.2 μg per seed, Figure 5A), explaining why the difference in phospholipid contents is only observed with HPTLC analyses. One mg of *era1-8* seeds contains slightly less TAGs than WT and *ggb-2* (Supplementary Figure 2C). However, although *era1-8* seeds are larger, one *era1-8* seed contains an equal quantity of TAGs as WT or *ggb-2* seeds (Figure 5B). We then investigated FA distribution in the 3 genotypes. Gas chromatography analysis reveals that *era1-8* has an altered FA distribution while *ggb-2* resembles to that of WT. Notably, *era1-8* seeds accumulate more C18:1 and C18:2, and display a lower C18:3 content (Figure 5C). Repartition of C18:0, C20:2 and C22:1 is also altered with less pronounced variations (Figure 5C). Furthermore, TAGs are enclosed within lipid bodies that consist of a monolayer of phospholipids and structural proteins, mainly steroleosin and oleosins (Jolivet et al., 2004). Consistent with the similar quantity of TAGs observed in the three genotypes, WT, *era1-8* and *ggb-2* seeds display comparable lipid body-associated protein patterns (Figure 5C, inset).

All these data indicate that protein farnesylation, but not geranylgeranylation, may control seed size determination and the production of seed storage compounds (i.e., protein content and FA distribution).

### Silique Development and Seed Production Are Altered in *era1-8*

**FIGURE 6:**
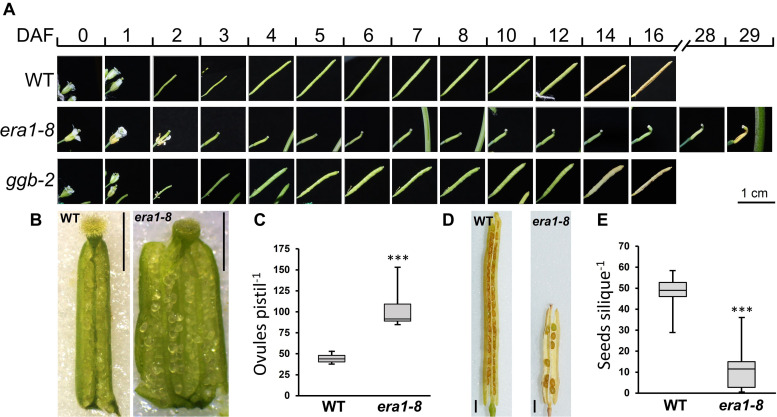
Silique development and seed production. **(A)** Kinetic of silique development of WT, *era1-8* and *ggb-2*. **(B)** Representative pictures of ovules within open ovaries of WT and *era1-8* at DAF0. **(C)** Quantification of ovules in WT and *era1-8* ovaries at DAF0 (Student’s *t*-test, *n* = 10). **(D)** Open mature siliques of WT and *era1-8*. **(E)** Quantification of seed production in WT and *era1-8* mature siliques (ANOVA, *n* = 30). DAF, Day after flowering. Scale bar in 6B and 6D is 1 mm. *** indicates a *p*-value < 0,001.

In Arabidopsis, pollination and fertilization follow the flower opening and then, embryo development and seed maturation take place. Until silique dehiscence, this process occurs within 16 days for WT and *ggb-2* plants (Figure 6A). Silique development is dramatically delayed in *era1-8*. At day four, whereas WT and *ggb-2* siliques start to elongate, *era1-8* siliques remain shorter and the tip starts to crook. Yellowing of siliques that corresponds to the end of the seed maturation, is observed at day 29 for *era1-8*, instead of day 16 for WT and *ggb-2*. Silique dehiscence is delayed by 13 days in *era1-8* (Figure 6A). Moreover, *era1-8* mature siliques are significantly smaller than WT and *ggb-2* (Figure 6A and Supplementary Figure 1), distorted and display a crooked tip (Figure 6A). Moreover, at DAF0, *era1-8* stigma does not display fully developed papillae as WT (Figure 6B). Under our growth conditions (i.e., short days), most of *era1-8* gynoecium are constituted by three carpels and develop numerous ovules compared to WT (Figure 6B). Variation in carpel number was observed in three other alleles of *era1* (i.e., *wig-1*, *wig-2*, and *wig-3* corresponding to *WIGGUM*, a former name of *ERA1*, Running et al., 1998), nevertheless this phenotype is more developed under short day growth conditions than long days (Yalovsky et al., 2000b). Quantification of ovule production reveals that *era1-8* produces about twice more ovules than WT (Figure 6C), which represents around 24 and 31 ovules per carpel for WT (2 carpels) and *era1-8* (3 carpels), respectively. Surprisingly, *era1-8* mature siliques contain few seeds (Figure 6D). WT plants produce frequently around 45–50 seeds per silique whereas it is highly variable in *era1-8* and the median production is restricted to 12 (Figure 6E).

## *era1-8* Produces Proper But Immature Ovules at Flower Opening

**FIGURE 7:**
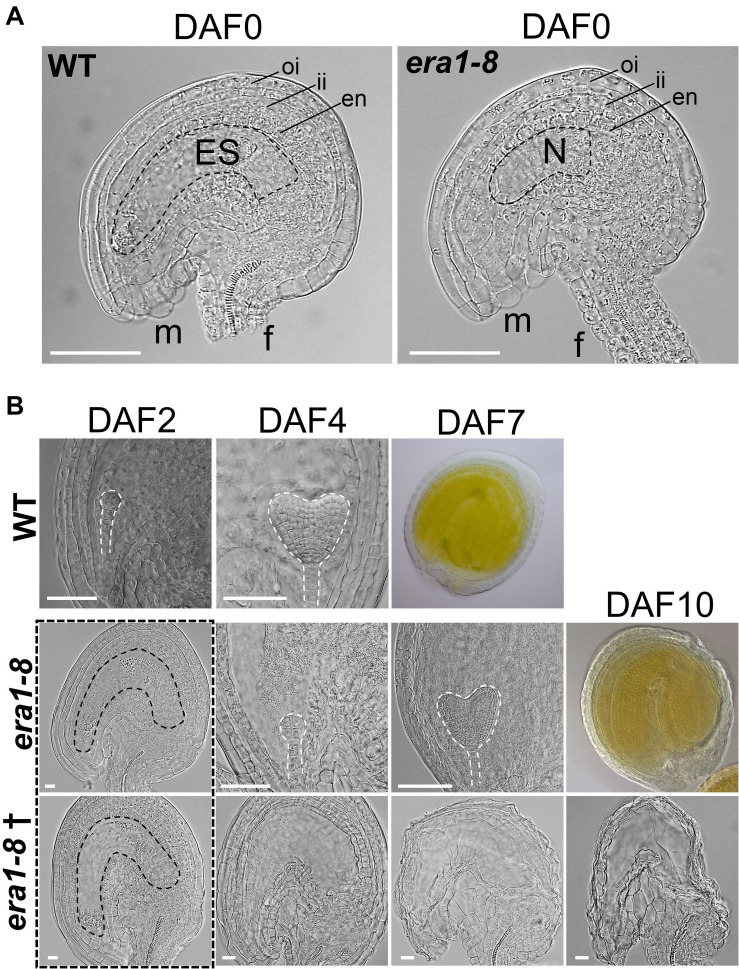
Comparison of WT and *era1-8* ovules. **(A)** Detailed views of DAF0 unfertilized ovules of WT (with embryo sac) and *era1-8* (without embryo sac). **(B)** Developmental kinetic of WT and *era1-8* embryos (dashed lines) at indicated time. † corresponds to unfertilized *era1-8* ovules. Dotted rectangle represented proper ovules observed at DAF2 in *era1-8* with different futures. oi, outer integuments; ii, inner integuments; en, endothelium; m, micropyle; f, funiculus; ES, Embryo Sac; N, Nucellus [according to Yu et al. (2005)]. Scale bar 250 μm in panel **(A)** and 50 μm in panel **(B)**. DAF, Day after flowering.

To understand why most of *era1-8* ovules do not develop into seeds, we scrutinized the fate of *era1-8* ovules at flower opening and the following days. Observations of ovules collected from WT and *era1-8* ovaries at flower opening (i.e., DAF0, Day after flowering #0) reveal that *era1-8* plants produce proper peripheral ovules tissues consisting of outer and inner integuments, endothelium, funiculus and micropyle as observed in WT (Figure 7A). However, *era1-8* embryo sac is not fully developed at DAF0 whereas WT ovule exhibits a large embryo sac (Figure 7A). At DAF2, no embryo is visible in *era1-8* ovules whereas WT ones already display globular embryos (Figure 7B). At DAF4 and DAF7, a developing embryo is visible in WT ovules at heart and green mature embryo stages, respectively (Figure 7B). In *era1-8* ovules, the globular embryo stage is observed at DAF4 and the heart stage at DAF7, the green mature embryo stage is reached at DAF10. Actually, embryo development from globular embryo stage to green mature embryo stage takes five to six days in *era1-8*, as observed for WT. This indicates that, once the ovules are mature (i.e., with embryo sac), after fertilization, *era1-8* embryo development is similar to WT. According to expression data (Figure 1A), *ERA1* expression level is higher in the globular stage and then deceases during the seed development, which suggests that protein farnesylation may be a determinant process for embryo early development. Moreover, as exemplified (Figure 7B, lane *era1-8*†, DAF2-10), although *era1-8* ovules display a fully developed embryo sac at DAF2, most of them do not develop embryo and end up degenerating (DAF10). This is consistent with the low seed production observed in *era1-8* siliques (Figures 6D,E).

### Pollination Efficiency May Be a Major Obstacle to *era1-8* Seed Production

**FIGURE 8:**
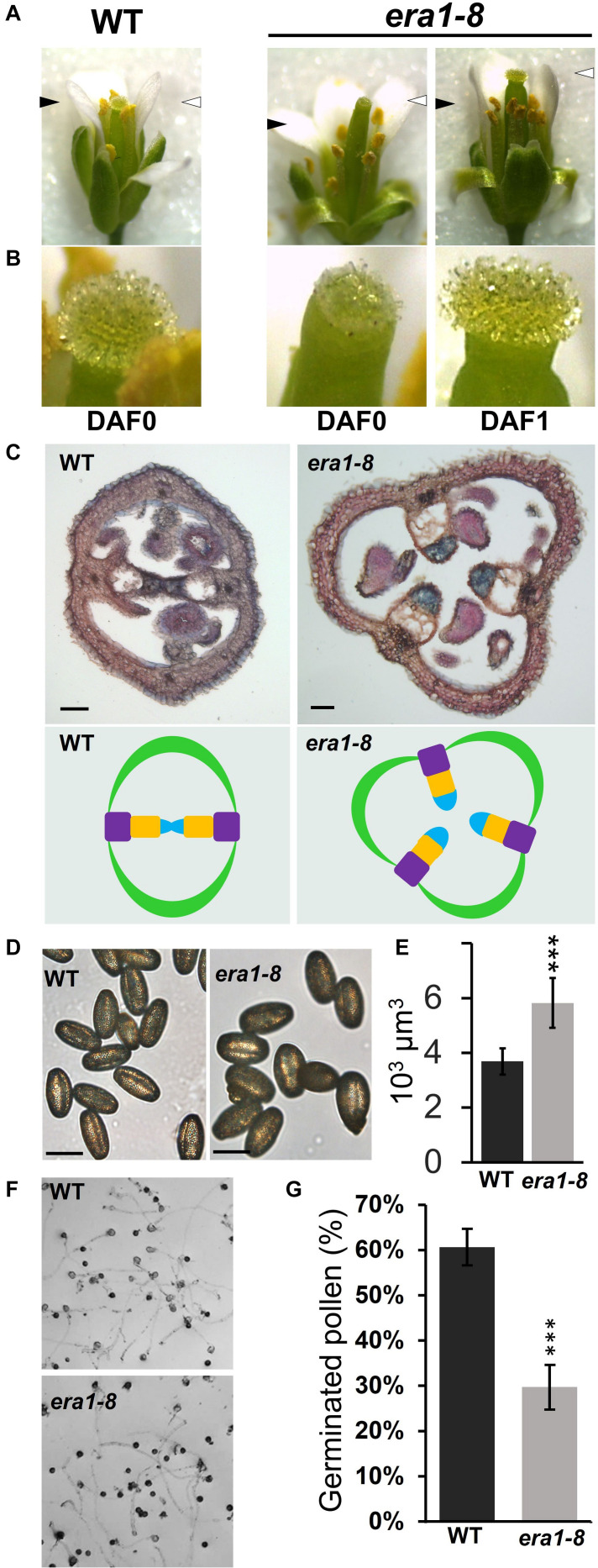
Gynoecium defects and pollination in *era1-8*. **(A)** Anther and stigma relative positioning at flower opening at indicated time. Black and white arrowheads indicate the top of stamens and stigmas, respectively. **(B)** Close-up views of flower’s stigmas and papillae shown in panel **(A)**. **(C)** Transversal cross-sections of young WT and *era1-8* siliques stained with neutral red and Alcian blue (scale bar, 50 μm). The corresponding ovary organization is drawn below (green, carpel; violet, replum; orange, septum; blue, transmitting tract). **(D)** WT and *era1-8* freshly harvested pollen (scale bar, 50 μm). **(E)** Estimated pollen volumes. **(F)** *In vitro* pollen germination assays. **(G)** Quantification of germinated pollen grains ± SE (Student *t*-test, *n* = 6). DAF, Day after flowering.

Because *era1-8* plant produces proper ovules that may be fertilized and able to develop embryos, ovule degeneration can arise from a deficiency in fertilization or a spontaneous abortion. We then investigated *era1-8* flower abilities to perform pollination. As previously described (Northey et al., 2016), *era1-8* flower exhibits a protruding pistil already observable in flower buds (Supplementary Figure 3), i.e., before anthesis. At flower opening (Figure 8A, DAF0), WT harbors two short stamens and four long stamens that are in contact with the stigma, which promotes the pollination. In this way, in the Arabidopsis Col-0 accession, fertilization further occurs mainly thanks to self-pollination (Nasrallah et al., 2004). In *era1-8* flowers, all stamens appear below the stigma (Figure 8A) and remain below after flowering. This should significantly reduce self-pollination and lower seed production. Moreover, at DAF0, papillae that are required for pollen recruitment on stigma are fully developed in WT (Figure 8B). A close-up view to *era1-8* stigma reveals that at DAF0, papillae are clearly shorter (Figure 8B, DAF0) and require one additional day to acquire a comparable appearance to those of WT (Figure 8B, DAF1). The delay in papillae expansion may thus further reduce pollen capture and pollination efficiency in *era1-8*.

Arabidopsis WT ovary is bilocular and consists of two valves (open carpels) connected by two replums. An additional transversal wall, the septum, links the two replums and supports the transmitting tract that facilitates pollen tube growth (Lennon et al., 1998). Because of callose deposition, Alcian blue specifically stains the transmitting tracts among ovary tissues (Reyes-Olalde et al., 2013). Cross sections of WT and *era1-8* siliques reveal that, in most cases, *era1-8* has 3-carpels ovaries with a disturbed organization (Figure 8C and Supplementary Figure 4A). The septum does not link the replums and the transmitting tracts appear free in the unilocular ovary (Figure 8C and Supplementary Figure 4A).

According to Bonetta et al. (2000), *era1* pollen release is not delayed but microsporogenesis is strongly perturbed in *era1*. Indeed, the microsporocyte meiosis is not synchronous and may produce aberrant tetrads and misshaped or degenerated microspores, reducing the quality of the pollen production. Although *era1-8* mature pollen grains display a comparable shape with that of WT (Figure 8D), they are significantly larger (Figure 8E and Supplementary Figure 4B). Pollen germination capacity, assessed through *in vitro* assays, highlighted that in our experimental conditions, WT pollen grains reach up to 60% germination whereas *era1-8* ones are limited to 30% (Figures 8F,G).

### Handmade Pollination Partially Restores WT Phenotype in *era1-8* Seeds

**FIGURE 9:**
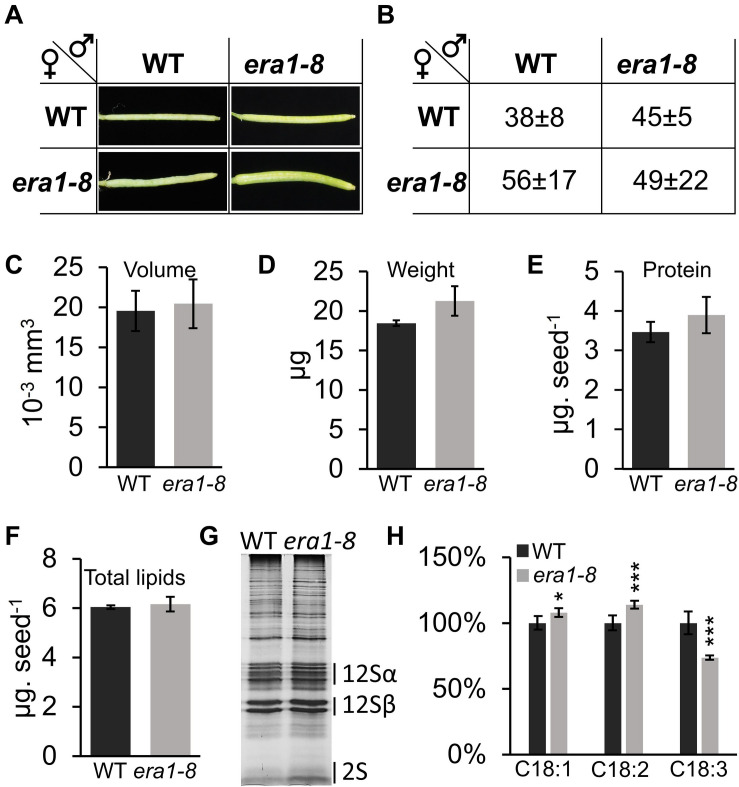
Phenotyping of seeds obtained after hand pollination. **(A)** Pictures of representative siliques obtained after handmade pollination with indicated crosses. **(B)** Average of seed number within siliques from (a) (*n* = 5 for WT and 20 for *era1-8*, see section “Materials and Methods”). **(C)** Volume of seeds obtained from WT x WT (WT) or *era1-8* x *era1-8* (*era1-8*) handmade crosses. **(D)** Seed weight. **(E)** Seed protein content. **(F)** Seed total lipids. **(G)** SDS-PAGE protein pattern. **(H)** Repartition (%) of indicated FAs. Error bars are ± SE.

In order to decipher whether pollination is critical for *era1-8* seed phenotypes, hand pollinations were performed on WT and *era1-8* flowers. Stamens where removed from flowers before opening and pollen was directly applied on pistils. As shown in Figure 9A, *era1-8* pistils pollinated by WT pollen produce siliques with a comparable shape of WT ones (WT × WT). A reciprocal cross (WT ovules vs *era1-8* pollen) achieves the same result. *era1-8* vs *era1-8* hand pollination restores a WT silique phenotype, as well, without a crooked tip (Figure 9A). This indicates distortion of *era1-8* tips during silique development (Figure 6A) is correlated with the low seed content. Besides, average seed production is partially restored regardless of the crossing made (Figure 9B). *era1-8* pollen applied on WT pistils leads to a seed production close to that of WT x WT hand crosses. Nevertheless, the pollination of *era1-8* pistils by either WT or *era1-8* pollens results in numerous non-developing seeds (Supplementary Figure 5) and *era1-8* show a highly variable seed production among the siliques (Figure 9B). Whatever the pollination method applied and the pollen genotypes, pollination and fertilization success of *era1-8* is difficult to control because of the delayed pistil maturity and its variable structural organization.

Morphology and biochemical characterization of seeds produced through *era1-8* x *era1-8* hand pollinations displays a near-WT phenotype: volume, weight, protein content and total lipid content were indeed restored (Figures 9C–F), suggesting that these characteristics rely on the number of seeds developing in the siliques. However, the lower 2S storage protein remains more abundant in *era1-8* x *era1-8* protein pattern (Figure 9G and Supplementary Figure 6A) and the distribution of C18:1, C18:2 and C18:3 is still altered, as observed when *era1-8* plants self-pollinate (Figure 9H and Supplementary Figure 6B). This further support the role of protein farnesylation in these biochemical seed traits.

## Discussion

Two decades ago, *ERA1* was involved in flower development thanks to thorough descriptions of *era1* mutants by Running et al. (1998) and Yalovsky et al. (2000a,b). The authors described enlarged floral meristems, late flowering, homeotic transformations of flowers and supernumerary organs in floral whorls. This organ number phenotype correlated with specific size changes in the early floral meristem and authors suggested that *ERA1* controls cell division and differentiation in the floral meristem. This would also explain why *era1* flowers often fail to develop (Yalovsky et al., 2000b). Besides *era1* floral phenotypes, our work highlights noticeable morphological and biochemical phenotypes of siliques and seeds that we specifically associated to the protein farnesylation (*era1-8*) but not to the protein geranylgeranylation (*ggb-2*). Overall, *ggb* plants are barely affected by the mutation (Johnson et al., 2005) compared to *era1* for which the phenotypes are probably based on specifically farnesylated CaaX-proteins. Nevertheless, this discrepancy can also be explained through the specific activities of PFT and PGGT-I. Indeed, PFT is less specific than PGGT-I for CaaX-box sequences, PFT can therefore compensate for loss of PGGT-I in *ggb* mutants more effectively than PGGT-I can compensate for loss of PFT in *era1-8* mutants (Andrews et al., 2010). Consequently, as described for vegetative developmental traits and the flower shape, seed production and quality are also more altered in *era1-8* than in *ggb-2*.

Nevertheless, some of the *era1-8* seed phenotypes that we describe can be, at least partially, attributed to CaaX-proteins cited in the literature.

### *era1-8* Flowers Produce a Low Amount of Seeds Despite Ovules Abundance

*era1-8* produces twice more ovules than WT plants. Although hand pollination improves *era1-8* seed production to a comparable level to that of WT, it does not lead to the full development of all *era1-8* ovules into seeds. So, *era1-8* low seed production can be attributed to both spontaneous ovule abortions and low self-pollination efficiency (Figures 8, 9). Indeed, a protruding pistil seems incompatible with self-pollination and it may explain the low seed production in *era1-8*. Northey et al. (2016) described protruding pistils in the flowers of *cyp85a2* mutants. Interestingly, *CYP85A2* gene encodes a CaaX-protein, belonging to the cytochrome P450 family, involved in brassinosteroid synthesis. Loss of either CYP85A2 or CYP85A2 farnesylation (mutated CaaX-box) resulted in reduced brassinolide accumulation (Northey et al., 2016). In *era1*, protruding pistils can thus originate from malfunctions of non-farnesylated CYP85A2. In addition, brassinosteroids are involved in various plant developmental process and play a major role in the control of pollen germination and growth (Ye et al., 2010; Vogler et al., 2014). Because *CYP85A2* is highly expressed in pollen tubes (Supplementary Figure 7A), we can speculate that involvement of CYP85A2 in reproductive traits may not only concern the protruding pistils but also concerns the pollen germination and/or tube growth. However, *era1* pollination faces several problems: protruding pistils, low germinating pollen grains, improper ovary transmitting tracts, delayed stigma papillae extension and delayed proper ovule formation (Figure 8). Because those traits are handled by different physiological pathways (Lord and Russell, 2002; Ma, 2005; Browse and Wallis, 2019), we can suspect that besides CYP85A2 several farnesylatable CaaX-proteins participate in the successful completion of pollination in WT Arabidopsis plants.

In addition to pollination defect, a closer view to hand pollinated *era1-8* siliques shows that numerous ovules do not develop into seeds, even when hand pollination is performed with WT pollen (Supplementary Figure 5). Indeed, WT and *era1-8* plants produce around 45 and 90 ovules per silique, respectively (Figure 6C). Hand-pollination of WT pistils with WT or *era1-8* pollens leads to the development of 38–45 seeds (Figure 9B) suggesting that *era1-8* pollen is efficient enough to ensure the fertilization by this way. When *era1-8* pistils are hand-pollinated, among the ∼90 ovules present in *era1-8* siliques, around 49–56 develop into seeds (Figure 9B and Supplementary Figure 5). According to our observations, it is difficult to assess whether non-developing seeds in *era1-8* result from (i) spontaneous ovule abortion, (ii) abortion due to no fertilization (because of pollen quality and/or carpel alterations), and (iii) ovule abortion at zygotic to pre-globular stages. Since the protein farnesyl transferase has hundreds of targets, in *era1-8*, non-developing seeds may result from overlapping malfunctions of CaaX-proteins involved in ovule/seed development. This may engage the *MEE56* (*Maternal Effect Embryo arrest 56*) gene, coding for a CaaX-protein, that we tracked in a list of 130 *loci* resulting from a large-scale screen of Arabidopsis mutants with defects in female gametophyte development (Pagnussat et al., 2005). MEE56 is the only CaaX-protein we identified in Pagnussat et al. (2005). Interestingly, *MEE* mutants may arrest embryo development at one-cell stage (i.e., zygote). They are also characterized by a slight general delay in embryo sac development and fertilization. Genetic approaches indicated these phenotypes rely on female gametophyte malfunctions (Pagnussat et al., 2005). MEE56 belongs to the HIPP (Heavy metal-associated Isoprenylated Plant Protein) family (45 members in Arabidopsis; MEE56 is HIPP18), which are all equipped with a C-terminal CaaX-box (de Abreu-Neto et al., 2013). Although HIPP molecular function has not been clearly established, isoprenylation affects their subcellular localization and protein-protein interactions (Cowan et al., 2018). *mee56* siliques bear some aborted seeds (Pagnussat et al., 2005), which may correspond to those observed in *era1-8* siliques that also rely on maternal traits (Supplementary Figure 5, ♀*era1-8* x ♂WT). According to Le et al. (2010) microarray experiments, *MEE56* expression is restricted to the chalazal endosperm (Supplementary Figure 7B), which allows the flow of nutrients from the phloem to the egg sac through the endosperm (Sager and Lee, 2014). A lack of farnesylation may disrupt MEE56 function or localization, and would lead to seed abortion in *era1*, as described in *mee56* siliques (Pagnussat et al., 2005).

The high ovule production observed in *era1-8* may be related to the increased number of carpels (Figures 6, 8). In Arabidopsis WT flowers, the number of carpels (i.e., two) is highly conserved whereas in the other whorls, the number of flower organs may be slightly variable (Running et al., 1998). In *era1*, supernumerary flower organs are often observed, including the carpel whorl (Running et al., 1998). Number of floral organs relies on floral meristem homeostasis that is determined by a complex interplay involving the *WUSCHEL*/*CLAVATA* (*WUS/CLV*) pathway, hormonal controls and dozens of genes (Somssich et al., 2016; Conti, 2017; Lee et al., 2019). Among these, the two homologs *AtJ2*/*AtJ3* (*A. thaliana* DnaJ homologue 2 or 3) encode CaaX-proteins that may partially explain *era1* floral-related phenotypes. Indeed, the *atj2*/*atj3* double mutant complemented with a non-farnesylatable form of *AtJ3* (*AtJ3*_*C417S*_) displays enlarged meristems similarly to *era1* and produces flowers with supernumerary petals. Nevertheless, only 16% of *AtJ3*_*C417S*_ flowers display supernumerary petals when it reaches 66% in *era1*, and no alteration of the number of carpels nor stamens has been reported in *AtJ3*_*C417S*_ (Barghetti et al., 2017). *WUS* is required for stem cell identity and *CLV* promotes organ initiation (Schoof et al., 2000). *clv* mutants harbor enlarged meristems and give rise to supernumerary stamens and carpels, but they maintain a WT-like sepal/petal number (Schoof et al., 2000). We can therefore suspect that, although farnesylation of AtJ3 is required for the determinism of petal number, *era1* floral phenotypes rely on other CaaX-proteins related to *WUS*/*CVL* pathway that control the determinism of the number of carpels. For instance, APETALA1 (AP1) is a well known transcription factor involved in the early floral meristem identity (Yalovsky et al., 2000a). *ap1* mutant develops flowers with carpeloid sepals and stamenoid petals. AP1 and its paralog CAULIFLOWER (CAL) have farnesylatable CaaX-boxes. Together, they participate in the transition of inflorescence meristem into floral meristem (Ye et al., 2016). *AP1* is mainly expressed during flower development but its expression is also detected in ovary as for *CAL* (Supplementary Figure 7C). *ap1*, *cal* and *ap1*/*cal* knock-out plants display more severe floral phenotype than *era1* suggesting that the non-farnesylated proteins present in *era1* flower maintain some functionality. Because, AP1 is also involved in flower organ number determination (Monniaux et al., 2018) and its actions are enhanced through CAL (Ye et al., 2016), the lack of farnesylation of both proteins may lead, in cooperation with AtJ2/AtJ3, to the abnormal number of carpels observed in *era1-8*. Investigating Arabidopsis transgenic *ap1/cal/AtJ2/AtJ3* plants co-expressing non-farnesylatable forms of AP1, CAL, AtJ2 and AtJ3 (i.e., mutated CaaX-boxes) with their specific transcriptional promoters may unravel the involvement of protein farnesylation in carpel number determination, nevertheless we cannot exclude that other unidentified CaaX-proteins make more complex the mechanism leading to this *era1-8* singular phenotype.

### *era1-8* Seeds Have Peculiar Biochemical Features

Interestingly, *era1-8* produces larger seeds than the WT, with different storage contents. They accumulate more proteins and have a different FA distribution. The control of seed size depends on genetic, environmental and physiological factors (Gnan et al., 2014; Orozco-Arroyo et al., 2015). Because hand pollination of *era1-8* flowers restores WT-like size and most of the biochemical phenotypes (Figure 9), the seed enlargement observed in *era1-8* may be a consequence of the low seed yield (Herridge et al., 2011). Nutrients provided by *era1-8* plants toward the flowers are distributed in few developing seeds, which consequently accumulate more storage compounds (Patrick and Offler, 2001; Zhang et al., 2007). Depending on mechanical constraints (Rolletschek et al., 2020), seed enlargement as well as silique impairments may also contribute to modify the accumulation of storage compounds observed in self-pollinated *era1-8* seeds. However, hand pollination do not restore the enhanced accumulation of 2S albumin (Figure 9G and Supplementary Figure 6A) nor the differential FA distribution observed in *era1-8* seeds (Figure 9H and Supplementary Figure 6B). Seed maturation involves master regulators such as ABI3, FUS3 (Fusca 3), LEC1 and LEC2 (Leafy Cotyledon 1 and 2) that govern embryo development and storage compound accumulation. It also involves hormonal regulations, mainly relying on ABA signaling (Baud et al., 2008; Kanno et al., 2010; Gao et al., 2012). Although the relation between ERA1 and ABA-signaling has not been fully elucidated, ABA enhanced sensitivity of *era1-8* may also perturb the control of storage compounds accumulation in seeds.

Beside an increased overall protein content in *era1-8* seeds, the 2S albumin accumulation is discernibly modified (Figure 4D). In Arabidopsis, five genes encode the 2S albumins (*At2S1-5*) (Gruis et al., 2002). Albumins are synthesized as precursors that are cleaved post-translationally by vacuolar processing enzymes (Otegui et al., 2006). Although the 2S albumin gene expression follows the embryo maturation process, albumins can accumulate differentially depending on nutrient intake (Higashi et al., 2006). For instance, sulfur modulates At2S3 accumulation but not its transcript level suggesting that albumin accumulation is regulated at the post-translational step rather than transcriptional level (Naito et al., 1994; Higashi et al., 2006). Furthermore, according to amino acid sequence analysis, the 2S albumin atomic composition is three time richer in sulfur compared to the 12S globin one, whereas the other atoms (i.e., C, H, N, and O) are similar (Supplementary Figure 8) which suggests that *era1-8* seeds have an overall enhanced sulfur content. Albumins are not farnesylated (no CaaX-box on precursors nor mature albumins; Shimada et al., 2003; Higashi et al., 2006), thus ERA1 action on albumin accumulation may stand on unidentified CaaX-proteins involved in nutrient perception or albumin post-translational cleavages during seed maturation.

Finally, *era1-8* seed phenotypes also deal with altered FA distribution. The major changes concern the increase of C18:1 and C18:2, and a decrease in C18:3 (Figure 5C). The C18:2/C18:3 balance (related to ω6 and ω3) is essential for human fitness and animal feed (Okuyama et al., 2007), and it became an important trait for seed oil selection. In Arabidopsis seeds, FA distribution relies on well-characterized regulatory network and biosynthetic pathway (Baud et al., 2008). These include ω-6-fatty acid desaturase2 (FAD2), ω-3-fatty acid desaturase3 (FAD3), fatty acid elongase1 (FAE1) and diacylglycerol acyltransferase1 (DGAT1) (To et al., 2012), which are critical for determining the composition and/or quantity of seed storage oil in Arabidopsis (Li-Beisson et al., 2013). C18:1, C18:2 and C18:3 distributions are tightly linked by their biosynthetic pathway: C18:1 desaturation involves FAD2 to produce C18:2 and then FAD3 to produce C18:3 (Baud et al., 2008). *FAD3* gene expression requires a transcriptional complex that engages L1L (Leafy cotyledon1-Like), NF-YC2 subunit and βZIP67 (Mendes et al., 2013). Interestingly, *l1l*, *nf-yc2* and β*zip67* mutants have an altered fatty acid composition and *l1l* C18:1/C18:2/C18:3 balance resembles to that of *era1-8*. None of these proteins are farnesylatable, nevertheless Dutilleul et al. (2016) described ASG2 (Altered Seed Germination 2), a farnesylated CaaX-protein involved in FA metabolism (Ducos et al., 2017). ASG2 belongs to a E3 ubiquitin ligase complex and interacts with histone deacetylase when farnesylated. Seeds of *asg2* mutants have a higher level of C18:1 and a reduced C18:3 content, suggesting that ASG2 may control *FAD2* and/or *FAD3* expression through histone modifications, as described for the histone acetyltransferase GCN5 (General Control Non−repressed protein 5) (Wang et al., 2016). Although the FA phenotype of *asg2* only partially overlap with *era1-8*, the lack of farnesylation of ASG2 might contribute to *era1-8* FA peculiarities.

Major agronomic traits, such as enlarged seed and protein content, rely on self-pollination deficiency and cannot thus be exploited for further agronomic purposes. However, quality of FA composition in *era1-8*, as well as, 2S albumins accumulation would disserve more attention to improve seed nutritional quality.

## Conclusion

**FIGURE 10:**
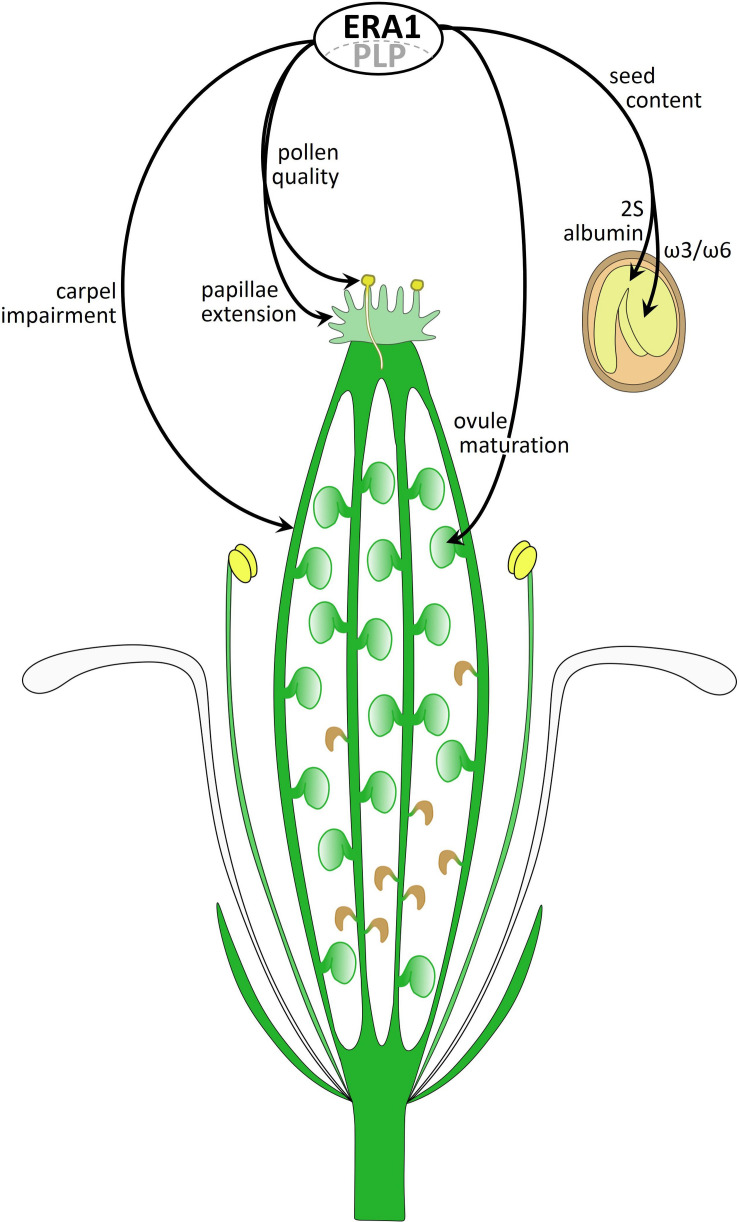
Diagram of an *era1-8* flower summarizing farnesylation-related phenotypes. ERA1 with the PLP α-subunit constitute the active farnesyltransferase that performs CaaX-protein farnesylation. The picture summarizes novel *era1* phenotypes involving unidentified CaaX-proteins.

Figure 10 summarizes new phenotypes developed by the *era1-8* mutant that we identified. Several phenotypes are related to gynoecium impairments: carpel number and architecture, delayed papillae extension, pollen quality and ovule maturation. Other phenotypes rely on the seed morphology (i.e., size, weight) and storage compounds (i.e., ω3/ω6 FAs, 2S albumins). We assume that these various phenotypes are the consequence of dysfunctions of several CaaX-proteins in *era1-8*. To date, most of the prenylated protein functions have been established through the characterization of knocked-out plants for the corresponding genes. Thus, the phenotypes observed do not correspond to a deficiency in isoprenylation but rather to a total absence of the functional protein in the plant, which is different of what happens in *era1*. Investigating the role of protein farnesylation requires the production of the non-farnesylatable protein in plant without the wild-type form. This can be achieved through the utilization of genome editing technologies allowing specific modifications of the CaaX-box encoding sequence for a unique farnesylatable protein. This strategy could lead to understand the role of isoprenylation on CaaX-protein behaviors and to decipher the *era1* intricate phenotypes.

## Materials and Methods

### Plant Material

Experiments were performed using *A. thaliana* accession Columbia (Col-0), the T-DNA insertion mutant *era1-8* [stock name SALK_110517 described in Goritschnig et al. (2008)] and the T-DNA insertion mutant *ggb-2* [stock name SALK_040904C described in Running et al. (2004)]. Plants were grown in a growth chamber (24°C at 70% humidity) under a 12 h light/12 h dark photoperiod and were exposed to an 80–100 μmol.m^–2^.s^–1^ irradiance. Once harvested, seeds were maintained in the dark at 4°C.

### Gene Expression Analysis

Expression data were retrieved from the Bio-Analytic Resource (BAR) Expression browser^2^ by querying the database with the Seed and Silique Development filter. Raw absolute expression data were centered and scaled per gene and the heatmap was generated with Excel software. Accession numbers: *ERA1* (*Enhanced Response to ABA1*), At5g40280; *GGB* (*GeranylGeranyl transferase Beta subunit*), At2g39550; *PLP* (*PLuriPetala*), At3g59380.

### Seed and Pollen Morphological Analyses

Length and width of 50 dry seeds from five independent biological replicates (i.e., five plants) for each genotype were determined using the ImageJ software^3^ and microscopy pictures. The volume of the seeds was calculated by equating the seed shape to a spheroid, according to Riefler et al. (2006). A similar approach was used to determine pollen grain sizes and volumes.

To determine the Arabidopsis seed weight, 500 dry mature seeds (stored in dark at 4°C for 2 months after harvest) were weighted with five independent biological replicates for each genotype with three technic replicates.

### Near Infrared Spectroscopy

NIRS method and predictive equations developed by Jasinski et al. (2016) were used to determine carbon, nitrogen, protein and lipid contents in Arabidopsis seeds. Seed samples (about 300 μl of seeds) were placed in a 9 mm diameter clear glass bottle (Agilent, 5182-0714) on 4 mm height for NIRS spectra acquisition and were analyzed as intact (without any treatment). Spectra acquisition was performed with a Fourier transform near-infrared (FTNIR) analyzer (Antaris II spectrometer; Thermofisher Scientific, France). Spectra were collected as described by Jasinski et al. (2016). The spectral data provide useful information about the organic signature of the Arabidopsis samples.

### Seed Protein Analyses

Seed total protein extracts were prepared as described in Rajjou et al. (2008) with minor changes. 50 dry mature seeds were hand-grinded using mortar and pestle at 4°C in 200 μL of extraction buffer consisting of 18 mM Tris-base, 14 mM Tris–HCl, 7 M urea, 2 M thiourea, 4% CHAPS, 0.2% Triton X-100, 1 mM PMSF (Harder et al., 1999). Samples were left on ice for 10 min. After the addition of 14 mM dithiothreitol, samples were incubated for 20 min at 4°C with shaking and clarified by centrifugation for 20 min at 20.000 *g* at 4°C. Protein concentration was determined in the supernatant using the Bradford method (Bradford, 1976). Protein extracts were analyzed by 12% SDS-PAGE and proteins were revealed by silver staining.

Proteins of seed lipid bodies were prepared from 25 mg of seeds using sucrose flotation techniques including successive NaCl, Tween 20 and urea treatments in order to remove non-specifically trapped proteins, as described by Jolivet et al. (2004). After an overnight acetone precipitation, dry pellets were dissolved in Laemmli sample buffer and directly loaded on 15% SDS-PAGE. Proteins were then stained by Coomassie blue procedure.

### Seed Lipid Analyses

Triacylglycerol (TAG) and total phospholipid quantifications were performed by High-Performance Thin-Layer Chromatography (HPTLC). Lipids were extracted by grinding 10 mg of dry seeds in 1 ml of dichloromethane:chloroform (2:1, v:v) 5 times during 1 min in a Mixer Mill MM400 (Retsch) at 30 hz. Samples were cooled on ice 30 s during each grinding cycle. Lysates were filtrated and lipid extract collected according to Bligh and Dyer (1959). Lipid extracts were spotted on HPTLC precoated silica gel glass plates (60F254 Merck, Darmstadt, Germany) using a CAMAG autosampler ATS4 sample applicator (CAMAG, Muttenz Switzerland). Before loading, plates were pre-developed once in chloroform:methanol (1:1, v:v), air-dried, and activated at 110°C for 20 min. Samples were applied in the form of 10-mm band shapes, 1.5 cm from the bottom of the silica plate at a constant flow rate of 150 nl.s^–1^ under nitrogen flow. Development was carried out with a mobile phase composed of hexane:ether:acetic acid (70:30:1, v:v:v). HPTLC plates were then air-dried for 90 min, and treated for 1 min in an immersion tank containing a 50% perchloric acid solution. After a 2-h drying period at room temperature, plates were heated for 16 min at 160°C to allow carbonization for the staining reaction. Densitometric analysis was performed using a TLC/HPTLC video-densitometer (TLC Visualizer 2 CAMAG, with Videoscan software, Camag, Muttenz, Switzerland). TAG and total phospholipids quantifications were performed using standard calibration curves obtained by spotting standards of known lipid composition.

For fatty acid analysis, TAG were separated by one-dimensional TLC (LK5, 20 × 20 cm, Carlo Erba, Val de Reuil, France) using hexane:diethyl ether:acetic acid (60:40:1, v:v:v) as mobile phase. TAG spots were scraped and collected in screw-cap glass tubes. TAG were prepared as fatty acids methyl esters before gas chromatography analysis (GC-2010plus, Shimadzu Scientific instruments, Noisiel, France) using a BPX70 capillary column (60 m, SGE, Chromoptic SAS, Courtaboeuf, France). Hydrogen was used as carrier gas with a constant pressure (120 kPa). After an on-column injection of sample at 60°C, oven temperature increased from 60°C to 220°C. Fatty acids methyl esters were detected by a FID at 255°C and identified by comparison of their retention times with commercial standards (Supelco 37, Fatty Acid Methyl Ester mix, Sigma-Aldrich, France, St Louis, MO, United States). Fatty acids levels were expressed as the percentage of total integrated peaks area using the GC Solutions software (Shimadzu, Noisiel, France).

### Arabidopsis Hand Pollination

For Arabidopsis crossing, one day prior to pollination, mature siliques, open flowers and buds having a white tip were removed from the plant. Immature buds were delicately opened with a needle under a binocular and stamens were removed with forceps. Two to three flowers were emasculated per inflorescence. After 1 day, stamens of freshly opened flowers of the desired plant were brushed against the emasculated flower stigmas. In order to ensure a maximum pollination rate, the pollination was repeated twice during 2 days on the same flowers after anthesis. Pollen presence on anthers was carefully checked with binocular magnifier.

For hand pollination of *era1-8* flowers, one stamen of a freshly opened flower was removed delicately and then brushed against the stigma of the same flower. The process was repeated until the presence of pollen on the stigma could be assessed under the binocular. Five experiments were conducted when WT pistils were used (*n* = 5 in Figure 9). Because *era1-8* pistils gave highly variable number of seeds, the experiment was repeated 20 times (*n* = 20 in Figure 9).

### Flower Observations and Ovule Clarification

Selected flowers were marked by a colored knot on the day of flowering and siliques were further collected at the desired day. Siliques were opened delicately under a binocular and ovules or developing seeds were incubated between microscopic slides overnight at room temperature in the dark in 50 μl of Hoyer clarification medium (24 g chloral hydrate, 3 ml glycerol and 9 ml Milli-Q^®^ water; Feng and Ma, 2017). Samples were then directly observed with an Olympus BX51 microscope equipped with a differential interference contrast (DIC) optic and an Olympus DP71 digital camera.

### Gynoecium Transverse Section and Transmitting Tract Staining

Flowers were incubated in FAA (50% ethanol, 5% acetic acid, and 5% formaldehyde) 16 h at 4°C. After ethanol and tert-butanol series, the samples were incubated overnight at 60°C, first in Paraplast^®^Plus:tert-butanol (1:1) and then in pure Paraplast^®^Plus (Leica Biosystems, Richmond, VA, United States). The Paraplast-embedded samples were sectioned to a thickness of 10 μm by using a rotary microtome. Sections were spread on slides pretreated with 2% 3-aminopropyltriethoxysilane (Sigma-Aldrich) in acetone (v:v), dried for 24 h at 40°C. Two 15-min incubations in xylene were used to remove paraffin from the samples, and an ethanol series up to water was used to rehydrate the sections. Sections were then stained for 2 h with Alcian blue (Sigma-Aldrich) to visualize acidic polysaccharides, such as callose, a major component of the transmitting tract (Crawford et al., 2007) and then with neutral red (to visualize cell walls) for 15 s. Samples were then observed on microscope.

### Pollen Germination Assays

Pollen germination assays were performed according to Li (2011). About ten open flowers (DAF1 to DAF3) for each genotype were collected and dried for 30 min at room temperature. After addition of 500 μL of germination medium (5 mM MES-Tris, 1 mM mM KCl, 0.5 mM MgSO_4_, 1.5 mM Boric acid, 10 mM CaCl_2_, 5% sucrose (w:v) and 15% PEG4000 (w:v), pH 5.8; Fan et al., 2001), flowers were shaken and pollen grains were collected by centrifugation at 500 *g* for 5 min. Pellet of pollen was suspended in 500 μl of germination medium. Pollen grains were germinated at 28°C overnight by placing 100 μl of the pollen suspensions in a 96 well plate (BioLite Thermo Fisher Scientific). Microscopy pictures of germinating pollen were obtained as described for ovules. The procedure was conducted six times for each genotype (*n* = 6).

## Data Availability Statement

The original contributions presented in the study are included in the article/Supplementary Material, further inquiries can be directed to the corresponding author/s.

## Author Contributions

VV and CD performed plant experiments, microscopy observations, and analyzed data. BG, BC, AL, and LR performed NIRS experiments and protein analyses. CG, MP, and SC performed seed lipid analyses. ED and CD planned and designed the research. CD, NG-G, and ED wrote the manuscript. All authors contributed to the article and approved the submitted version.

## Conflict of Interest

The authors declare that the research was conducted in the absence of any commercial or financial relationships that could be construed as a potential conflict of interest.

## Footnotes

1 http://bar.utoronto.ca/

2 http://bbc.botany.utoronto.ca

3 https://imagej.nih.gov/ij/
