# Supplementary material for: Protein Farnesylation Takes Part in Arabidopsis Seed Development
Source: Front Plant Sci. 2021 Jan 28;12:620325. doi: 10.3389/fpls.2021.620325 (PMC7876099; doi:10.3389/fpls.2021.620325)
Supplement: Supplementary file 1 [file Data_Sheet_1.PDF]

Supplemental Data

Figure 1

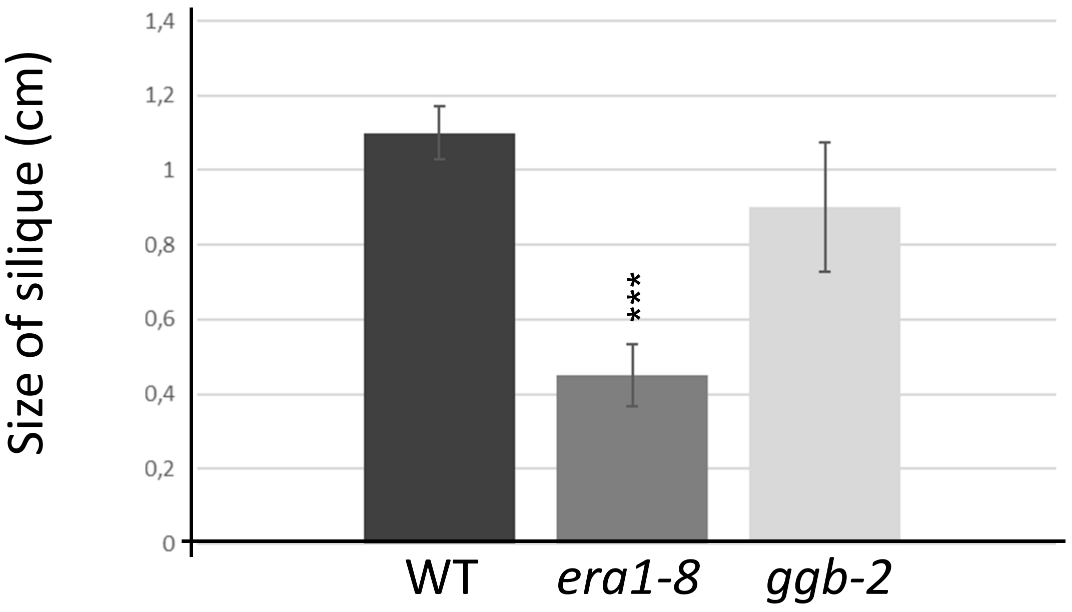

Fig. 1: Silique size measurement of WT, *era1-8 and ggb-2* plants. *** P value < 0.001 (Student's *t-test).*

Figure 2

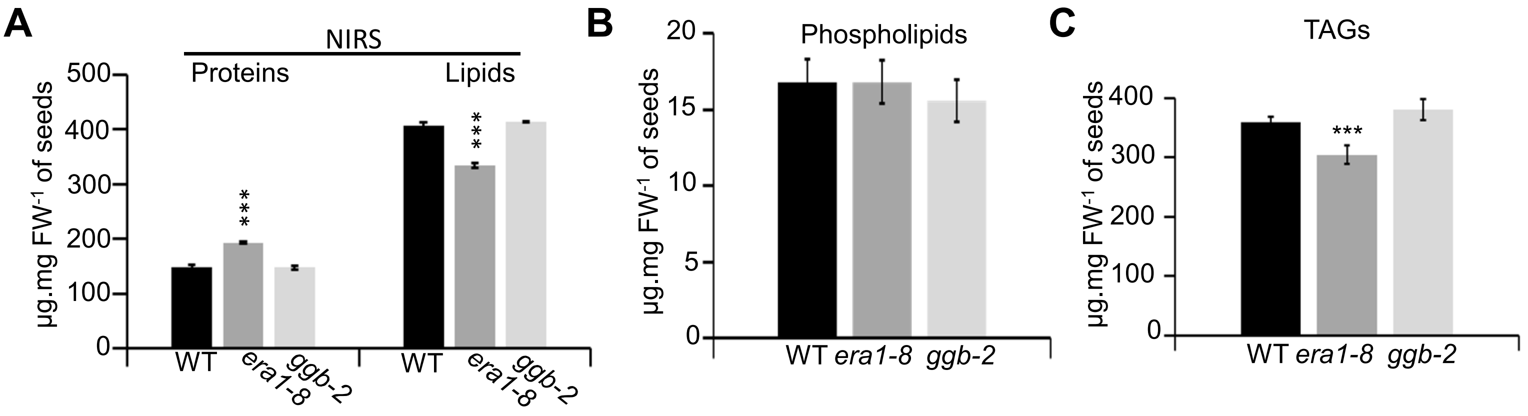

Fig. 2: **Proteins and lipids content expressed per mg of seeds.** (A) Results of NIRS analyses. (B) Total phospholipids contents per mg of seeds. (C) TAG contents per mg of seed. Data represent mean ± SE. *** p-value < 0.001 (Student's *t*-test). FW, Fresh weight.

Figure 3

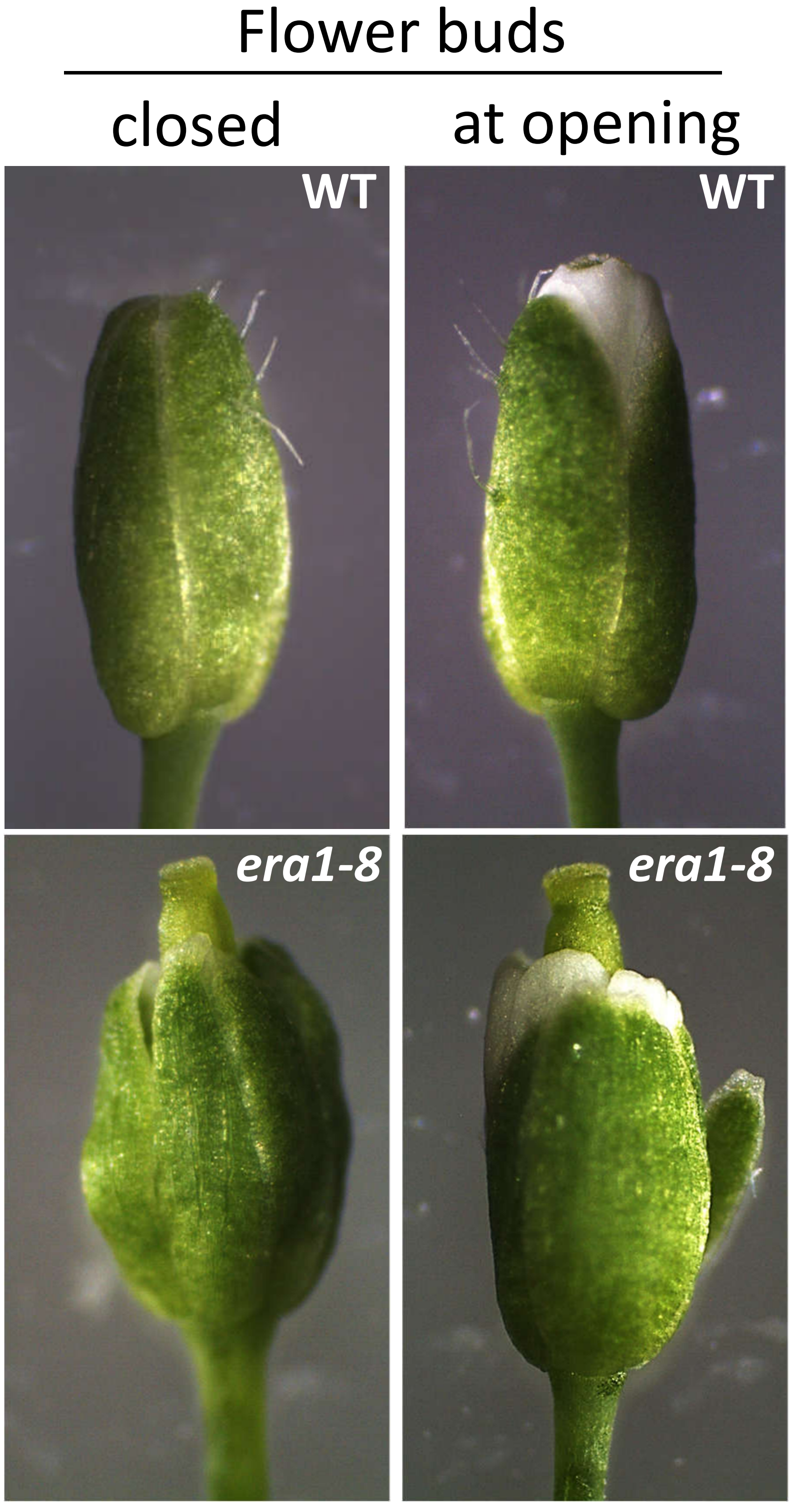

Fig. 3: Flower buds of WT and *era1-8* showing protruding pistils of *era1-8* before and at flower opening (visible petals).

Figure 4

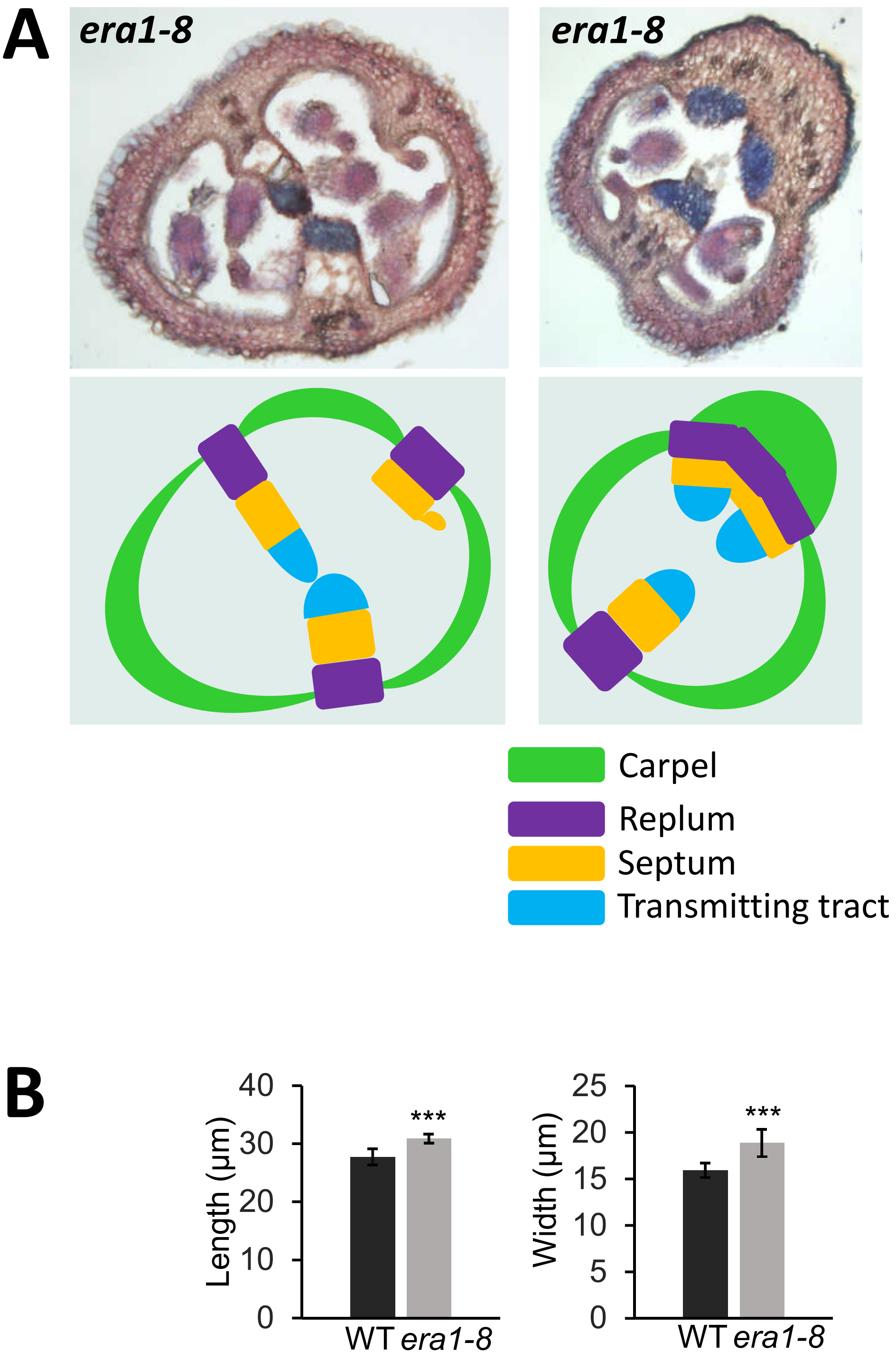

Fig. 4: Ovary organization and pollen grain measurement. (A) A photo gallery of *era1-8* 3-carpels ovaries with their representative diagram. Tissues are stained with neutral red and alcian blue. (B) Length and width measurement of WT and *era1-8* pollen used to calculate pollen volume in Fig. 9E.

Figure 5

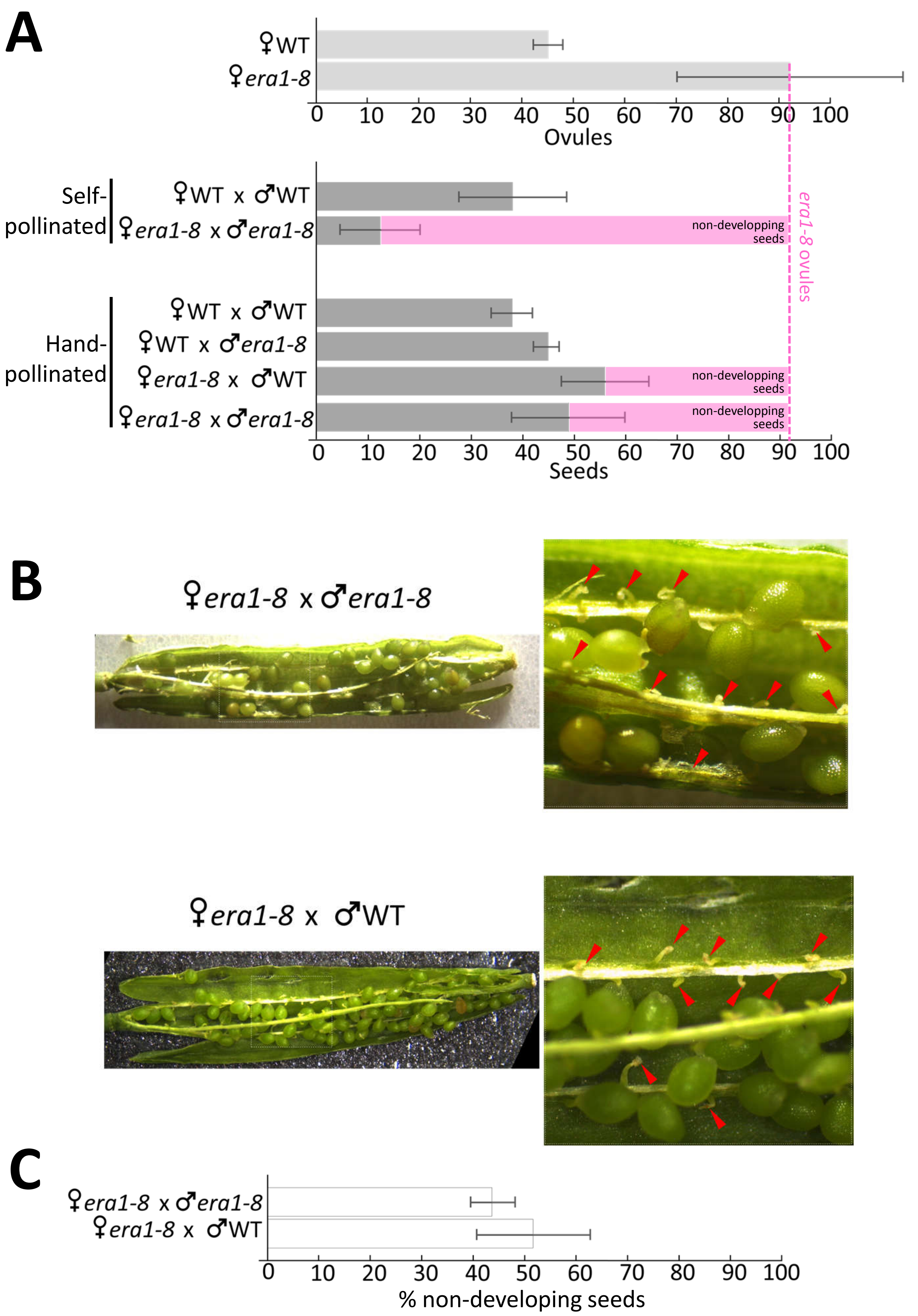

Fig. 5: Quantification of *era1-8* seed production and non-developing seeds. (A) Graphs summarizing data presented Fig. 6C (ovules), Fig. 6D (seeds, self-pollination) and Fig. 9B (seeds, hand-pollinated) with estimated *era1-8* non-developping seeds (pink). (B) Dissection of siliques obtained after handmade pollination with the indicated crosses (i.e. *era1-8* pistils with *era1-8* or WT pollen). On the right, closer views showing aborted ovules (red arrowheads). (C) Quantification of non-developing seeds in the indicated hand-pollinated crosses (n=20).

Figure 6

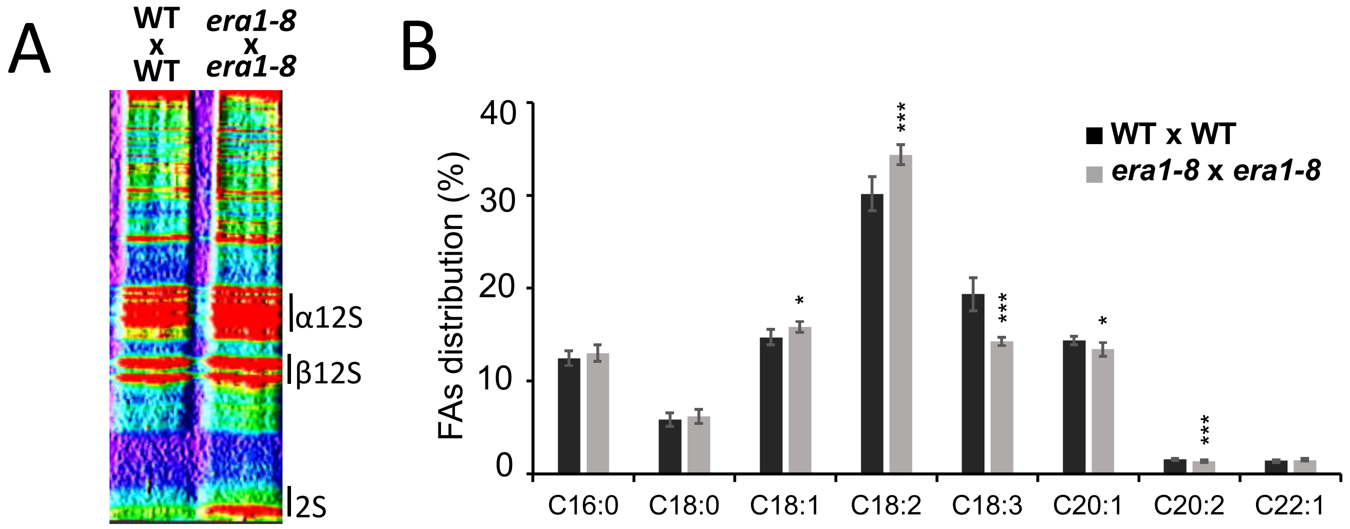

Fig. 6: Seed contents after hand pollination. (A) 15% SDS-PAGE of seed total protein extracts. The picture of silver-nitrate stained gel of the Fig. 9G was recolored with the ImageJ interactive surface plot Plugin. (B) Total distribution of FAs shown in Fig. 9H.

Figure 7

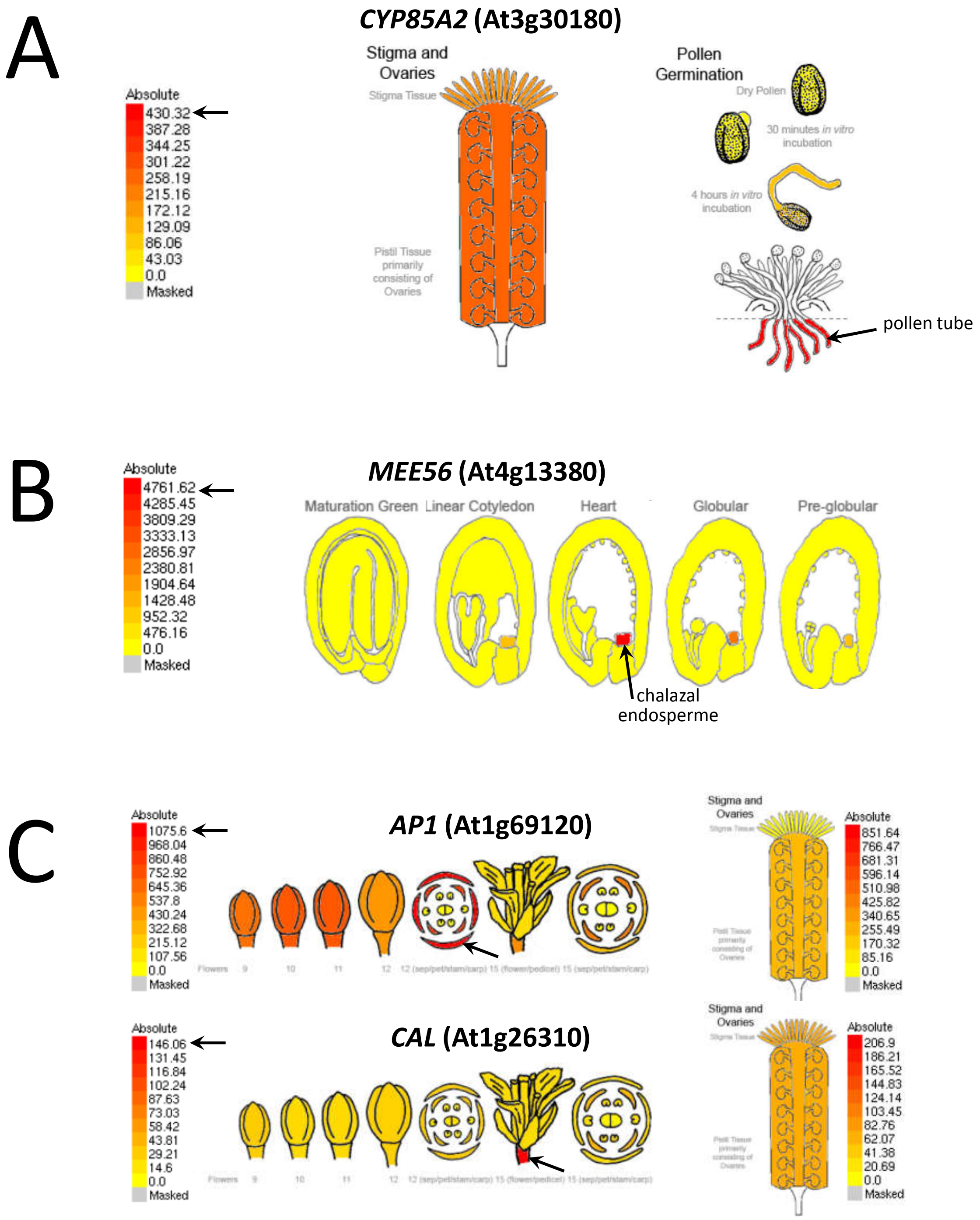

Fig. 7: Screen shots of the BioArray Ressource Arabidopsis eFP Browser (http://bar.utoronto.ca/) for indicated CaaX-protein encoding loci. (A) *CYP85A2* expression pattern recovered from the Tissue Specific data source. (B) *MEE56* expression pattern recovered from the Seed data source. (C) *AP1* and *CAL* expression pattern recovered from the Developmental Map and Tissue Specific data sources. Arrows indicate maximum expression level reach in a data source.

Figure 8

%	At2S1	At2S2	At2S3	At2S4	At2S5	CRA1	CRB/CRU2	CRC/CRU3	CRD
C	31,481	31,528	31,855	31,530	31,279	31,257	31,612	31,413	31,868
H	49,486	49,496	49,325	49,643	49,886	49,605	49,625	49,377	49,349
N	8,984	9,003	8,986	9,057	8,942	9,479	9,099	9,377	8,921
0	9,478	9,414	9,333	9,245	9,361	9,569	9,438	9,661	9,673
S	0,571	0,560	0,501	0,526	0,533	0,090	0,226	0,173	0,188

Percentage of atomic composition of individual 2S albumins (pink) and 12S globins (blue)

%	At2S+At12S	At2S	At12S	At2S/At12S
C	31,536	31,534	31,537	1,000
H	49,532	49,567	49,489	1,002
N	9,094	8,994	9,219	0,976
0	9,463	9,366	9,585	0,977
S	0,374	0,538	0,169	3,179

Average (percent) of atomic composition of 2S albumins (pink) and 12S globins (blue). At2S/At12S ratio is significantly different (black).
